# Children with pertussis inform the investigation of other pertussis cases among contacts

**DOI:** 10.1186/1471-2431-7-21

**Published:** 2007-05-22

**Authors:** Paulo N Baptista, Vera S Magalhães, Laura C Rodrigues

**Affiliations:** 1 Pediatric Infectious Diseases Service, School Hospital, University of Pernambuco, Recife, Brazil; 2 Tropical Medicine Department, Federal University of Pernambuco, Recife, Brazil; 3 Department of Infectious and Tropical Diseases, London School of Hygiene and Tropical Medicine, University of London, London, UK

## Abstract

**Background:**

The number of reported pertussis has increased in the last two decades. However, many cases of pertussis may be underreported or not diagnosed. The World Health Organization estimates that pertussis causes 200.000 – 400.000 deaths each year, most deaths are in infants and in developing countries. Infants with pertussis can indicate an undetected source cases in the community.

**Methods:**

At a University Hospital in Brazil individuals that had frequent contacts with a child with confirmed pertussis (the index case) and had recent history of cough were enrolled into the study. Nasopharyngeal swabs were collected from every contact that had cough within the last 21 days. Cases confirmation followed the guidelines of the Center for Disease Control and Prevention – Atlanta, U.S.A.

**Results:**

Pertussis diagnosis was confirmed in 51 children, (considered the index cases). Among the index cases, 72.5% (37/51) were under 6 months of age; culture for *Bordetella pertussis *was positive in 78.4% (40/51). Pertussis was confirmed in 39% (107/276) of the contacts of 51 index cases. Among these contacts identified as a pertussis case, 40.2% (43/107) were between 6 months and 11^1^/_2 _years of age and 59.8% (64/107) were older than 11^1^/_2 _years of age. Pertussis was confirmed by culture in 11.2% (12/107) of them and by epidemiologic linkage in 88.8% (95/107). Each index case allowed identifying two new cases of pertussis.

**Conclusion:**

Public health authorities should consider implementing early recognition of pertussis index cases and searching for pertussis cases among the contacts. Treatment of the cases and prophylaxis of the contacts is fundamental to control outbreaks in the community.

## Background

Pertussis is an endemic disease, with peaks every three to four years worldwide. Incidence of pertussis has declined, however many cases of pertussis may be underreported or not diagnosed. The World Health Organization estimates that 200.000 – 400.000 deaths occur each year, most deaths occur in infants and in developing countries [1]. Pertussis in infants younger one year of age should be considers an indicator of undetected disease in the community [2].

The transmissibility period of an individual with pertussis is around 21 days, but some can transmit for a longer period. Suspect cases and cases of pertussis should be isolated from young children, infants, child care centers, schools and public gatherings. The transmissibility period can be decreased and isolation ended after the fifth day of anti-pertussis antibiotic therapy [1]. Adolescents and adults have an important role as source of pertussis infection, particularly for these younger than one year of age [3,4]. In this age group, pertussis is frequently severe and hospitalization is often required [5]. Pertussis diagnosis among adolescents and adults is often missed or mistaken as another respiratory disease. In the last years, increased awareness of pertussis among adolescents and adults and more sensitive diagnostic tests (like serology and polymerase chain reaction assays) have contributed to increase the number of confirmed pertussis in this age group [6,7].

In Brazil until 2004, pertussis whole cell vaccine was given at 2, 4 and 6 months of age and a booster dose was given between 15 and 18 months of age. In the study area, pertussis vaccine coverage for 3 doses among children younger than one year of age was higher than 96%. The study of vaccine effectiveness in contacts revealed a low vaccine effectiveness against clinical disease, 12.5% (compared to children who received one dose only). On the other hand, vaccine effectiveness in reducing the transmissibility of a vaccinated primary case aged 7 months to 5 years old was 61.6%. This may have a significant effect in reducing circulation of *B. pertussis *in the studied population [8].

In 2003, in the Oswaldo Cruz School Hospital – Brazil, we followed the guidelines of the Center for Disease Control and Prevention – Atlanta, U.S.A. for pertussis diagnosis. [9]. Pertussis was confirmed in 158 individuals. In this year, most of children with pertussis were infants younger one year of age. Pertussis in infants younger one year of age should be considers an indicator of undetected disease in the community [2]. The objective of this study was to identify individuals with pertussis among the contacts of children with confirmed pertussis.

## Methods

In 2003, in the Oswaldo Cruz School Hospital (a metropolitan sentinel surveillance hospital for infectious diseases), children referred to the hospital with suspected pertussis and that had this diagnosis confirmed were identified as index cases. Children were considered a suspected case of pertussis if they had a cough illness lasting at least 7 days, with either paroxysms of coughing, inspiratory "whoop", post-tussive vomiting or cyanosis during the paroxysms of coughing without other apparent cause. Household members, neighbors and relatives that had frequent contacts with the index cases were enrolled into the study. Parents/guardians were interviewed, and information collected for the contacts of the index cases on recent history of cough. Every contacts of a confirmed case of pertussis with recent history of cough were requested to attend to the hospital.

Nasopharyngeal swabs were collected from every index cases and every contacts of the index cases that had cough within the last 21 days and had attended to the Hospital. Culture procedure followed the guidelines of the Centre for Disease Control and Prevention – Atlanta, U.S.A [10]. Clinical specimens were placed in Regan Lowe medium and incubated at 35°C to 37°C. Cultures were checked to up 12 days. *Bordetella pertussis *culture was confirmed by biochemistry test. Cultures were not collected from subjects with no reported symptoms. Although asymptomatic carriers exist, they are not relevant for transmission [11].

The household members, including the case attending the Oswaldo Cruz School Hospital, were classified: index case – the case that was first reported to the hospital; primary case – first individual in the household that presented cough and had pertussis diagnosis confirmed; co-primary case – had symptoms onset within 6 days after the onset in the primary case; secondary case – had symptoms onset 7 or more days after onset in the primary case; Not a case of pertussis – contacts without pertussis symptoms [9].

The index cases and their contacts were considered confirmed cases of pertussis if they had one of: **1**. an illness with cough of any duration and positive culture for *B. pertussis*; **2**. an illness with cough lasting at least two weeks and at least one of the following symptoms: paroxysm of coughing, inspiratory whooping, pos-tussive vomiting, a negative culture but a epidemiologic link to a culture confirmed case; **3**. an illness with cough lasting at least 14 days, in a household with two or more pertussis cases, where there were one culture confirmed case (a household outbreak).

Statistical analysis: Statistical significance was assessed using chi square (Fisher exact test when the value of one of the cells in a table was <5). A confidence interval of 95% was calculated and "p" was considered significant when ≤0.05. The study was approved by the Ethics Committee of HUOC. Parents/guardians signed a written consent form to participate.

## Results

Among 287 children assisted for suspect pertussis in the Pediatric Infectious Disease Service in the HUOC, pertussis diagnosis was confirmed in 51 (the index cases).

All index cases were under 11^1^/_2 _years of age, 92.2%(10/51) were under 5 years and 72.5% (37/51) were under 6 months of age. Among the contacts of the index cases that were identified as a pertussis case, 40.2% (43/107) were between 6 months and 11^1^/_2 _years of age and 59.8% (64/107) were older than 11^1^/_2 _years of age.

Pertussis vaccine report was obtained from the index cases: 72.5%(37/51) were not vaccinated or had received <3 doses and 25.5%(13/51) had received 3 or 4 doses of the vaccine. One patient didn't have information on his vaccine schedule. The difference between the group that was unvaccinated or received < 3 doses and these that received 3 or 4 doses was statistically significant. P < 0.01

Culture of nasopharyngeal swab was positive for *Bordetella pertussis *in 78.4% (40/51) of the index cases and negative in 19.6% (10/51) of them. One index case was in use of erythromycin for more than 5 days and swab for culture was not collected. All index cases met the clinical case definition for pertussis. Pertussis was confirmed by culture in 78.4%(40/51) of index cases and by epidemiologic linkage in 21.6% (11/51). Among the contacts of the index cases that were identified as a pertussis case, pertussis was confirmed by culture in 11.2%(12/107) and by epidemiologic linkage in 88.8%(95/107). A quarter of pertussis cases identified by the index cases were confirmed by outbreak case definition in household settings.

Twenty-two contacts were excluded because information about them was insufficient to classify them into cases or not cases. Two parents report relatives with cough who did not attend to the hospital. Some parents reported neighbors with history of cough, but they did not attend to the hospital as they were not household contacts.

**Figure 1:**
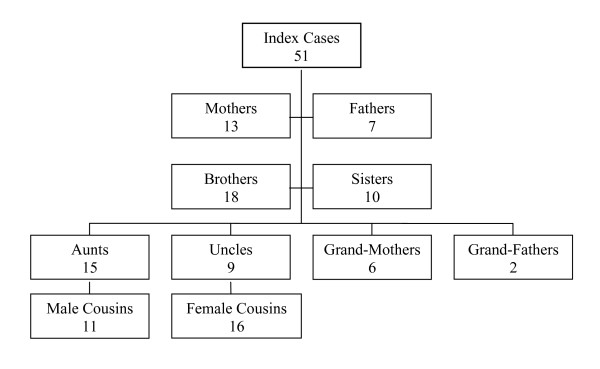
Pertussis cases among relatives of the index cases.

All the contact cases identified by the index cases were relatives (figure 1). Among the contacts, 56%(60/107) of the pertussis cases were women and 44%(47/107) were man chi^2 ^3,68 P = 0,05. Many relative were living in the same household, the same street or the same neighborhood. The ones that were living in different neighborhoods used to spend the week-end together in the same household.

Everyone who have had contact with the index case and did not have cough when examined received chemoprophylaxis. The index cases and secondary cases received treatment to pertussis as indicated by the Brazilian Health Ministry. Among 298 household contacts, pertussis was confirmed in 107 subjects. Each index case allowed identifying 2 new cases of pertussis. (Table 1)

## Discussion

In this study, nasopharyngeal culture was the only laboratory test used to confirm the diagnosis. It is likely that more cases would have been confirmed if serology and polimerase chain reaction assays were available. Finally, it is possible that some individuals in which the pertussis was confirmed using the "household outbreak criteria" (cough lasting at least 14 days, in a household with two or more pertussis cases, where there was one culture confirmed case) may have had another respiratory disease and have been misclassified.

This study was achieved in a service of pediatric infectious disease. This is likely to have contributed to the fact that all index cases were under 11^1^/_2 _years of age. However, children, particularly infants under one year of age frequently have severe pertussis and often require medical assistance [5]. This may in part explain the observation that three quarters of the index cases were under six months of age. This may also have contributed to the extremely high compliance resulting in almost all relatives of the index cases attending the hospital.

In the study, all adults with confirmed pertussis have presented symptoms that suggested the diagnosis of pertussis. Adolescents and adults are important source of pertussis infection for infant and even for others adults [3,4,12]. In the study, the primary case – the source of infection to the household – was older than 11 and a half years for 79% (66/84) of all secondary cases and for 76%(19/25) of the secondary case under 6 months of age [3]. Pertussis in a child under one year of age informs to investigate other pertussis cases among contacts [2].

Culture for *B. pertussis *has a low specificity for pertussis diagnosis and is often negative among vaccinated individuals and adults with Pertussis [10]. The fact that three forth of the index cases were unvaccinated or incompletely vaccinated may have contributed to the observation of positive culture in 78% of them.

Each index case in this research allowed the diagnosis of two other pertussis cases among the close contacts. The fact that the index cases and secondary cases were treated with antibiotics and the contacts without cough received chemoprophylaxis may have reduced the spread of pertussis among the contacts.

Public health authorities should consider implementing case finding in the community, particularly among infants younger one year of age, an important indicator of undetected cases in the community. Ideally, in addition to nasopharyngeal culture for *B. pertussis*, a more sensitive diagnosis test like PCR should be introduced to optimize the identification of new cases.

## Conclusion

Health care professional should be in alert to identify new cases of pertussis. The early recognition of pertussis index cases by health care professionals, the treatment of the cases and prophylaxis of the contacts is fundamental to control an outbreak in the community.

## Competing interests

This research received grants from the Pan-American Health Organization/World Health Organization

## Authors' contributions

P.N. Baptista and V. Magalhães conceived the study and supervised all aspects of its implementation. L. C. Rodrigues synthesized analyses and led the writing.

## Pre-publication history

The pre-publication history for this paper can be accessed here:



## Figures and Tables

**Table 1:** Classification of the index cases and of cases identified by index case in Oswaldo Cruz School Hospital in Brazil.

	Classification			
	Primary	Co-primary	Secondary	Total
	
Index cases	18 (35.2%)	2(4%)	31(60.8%)	51
Cases identified by index case	39(36.4%)	6(5.6%)	62(58%)	107
Total	57	8	93	158
